# Preventing SARS-CoV-2 In-Hospital Infections in Cardiovascular Patients and Medical Staff: An Observational Study From the German Heart Center Berlin

**DOI:** 10.3389/fmed.2020.616648

**Published:** 2021-02-03

**Authors:** Doreen Schöppenthau, Karl Jakob Weiß, Misael Estepa-Martinez, Matthias Hommel, Oliver Miera, Felix Schoenrath, Sabine Hübler, Martin Obermeier, Burkert Pieske, Philipp Stawowy

**Affiliations:** ^1^Department of Internal Medicine - Cardiology, Deutsches Herzzentrum Berlin, Berlin, Germany; ^2^DZHK (German Center for Cardiovascular Research), Partner Site Berlin, Berlin, Germany; ^3^Deutsches Herzzentrum Berlin, Department of Cardioanaestesiology and Intensive Care, Berlin, Germany; ^4^Department of Congenital Heart Disease - Pediatric Cardiology, Deutsches Herzzentrum Berlin, Berlin, Germany; ^5^Department of Cardiothoracic and Vascular Surgery, Deutsches Herzzentrum Berlin, Berlin, Germany; ^6^Deutsches Herzzentrum Berlin, Berlin, Germany; ^7^Laboratory MVZ MIB AG (Medizinisches Versorgungszentrum - Medizinisches Infektiologiezentrum Berlin AG), Berlin, Germany; ^8^Department of Cardiology, Campus-Virchow, Charité Universitätsmedizin Berlin, Berlin, Germany

**Keywords:** COVID-19, SARS-CoV-2, prevention, health care worker, face mask, nosocomial infection, in-hospital transmission

## Abstract

**Objective:** COVID-19 is a highly contagious disease caused by severe acute respiratory syndrome coronavirus 2 (SARS-CoV-2). Preventing in-hospital infections is crucial to protect patients and hospital staff.

**Methods:** At the very beginning of the COVID-19 pandemic, the German Heart Center initiated obligatory wearing of surgical face masks for patients and employees, SARS-CoV-2 screening for all patients, and symptom-based testing for employees. In addition, access restriction, closure of outpatient departments, and postponing non-urgent procedures were implemented with community-initiated regulations.

**Results:** During the observation period (03/16/2020–04/27/2020), 1,128 SARS-CoV-2 tests were performed in 983 persons (1.1 tests/person; 589 in patients and 394 in hospital employees). Up to 60% of the clinical workforce was tested based on symptoms and risk (62.5% symptoms, 19.3% direct or indirect contact to known COVID-19, 4.5% returnee from risk area, 13.7% without specific reason). Patient testing for SARS-CoV-2 was obligatory (100% tested). The overall prevalence of positive tests during the observation period was 0.4% (*n* = 5 out of 1,128 tests performed). The incidence of new infections with SARS-CoV-2 was 0.5% (*n* = 5 out of 983 individuals; three healthcare workers, two patients). No nosocominal infections occurred, despite a mean number of 14.8 in-hospital contacts.

**Conclusion:** Comprehensive SARS-CoV-2 testing and surgical face masks for patients and hospital staff, in addition to others measures, are key factors for the early detection of COVID-19 and to prevent spreading in the vulnerable hospital population.

## Introduction

Clustering of a severe acute respiratory distress syndrome was first described in Wuhan, China, in December 2019, with the subsequent identification of the coronavirus SARS-CoV-2 (severe acute respiratory syndrome coronavirus 2) as the causal agent of a disease now termed COVID-19 (coronavirus disease 2019) (1). COVID-19 is a highly contagious lower respiratory tract infection mostly transmitted via droplets, but airborne transmission was also reported (2, 3). Cardiovascular risk factors and cardiovascular complications during the course of the infection are important disease modifiers, contributing to a higher mortality (4–6). As of November 17, the number of infected patients exceeds 55.4 million globally, causing a death toll of more than 1,300,000 (7). In Germany, the first COVID-19 patient was reported in the southern state of Bavaria on January 27, 2020 (8), whereas the first case in the northern state of Berlin was reported on March 1, 2020 (9). Thereafter, the number of infected patients increased rapidly, reaching 817,526 in Germany up to date with 12,833 deaths (7). In several countries, the COVID-19 pandemic has led to an overwhelming demand on intensive care beds and ventilator therapy.

Infectiousness in the early stage of the disease and transmissions in the presymptomatic state or from persons with an asymptomatic course of the disease is likely high (10–12). This has been shown to cause clusters in vulnerable population, such as residents of nursing homes, as well as hospitalized patients (13, 14). Likewise, caretakers and healthcare workers (HCWs) are at increased risk of SARS-CoV-2 infection (15, 16).

Based on initial reports, a concept of strict compartmentalization between designated COVID-19 and non-COVID-19 hospitals has been recommended to prevent in-hospital transmissions (17). The University Hospital Charité and the state senate of Berlin established a 3-level model to ensure the distribution and care of COVID-19 and non-COVID-19 patients (“SAVE-Berlin/Brandenburg@COVID-19”) (18). Within this network, the University Hospital Charité is the level I center primarily responsible for the coordination and the treatment of severe cases. Additionally, there are 16 level II centers for COVID-19. In contrast, level III centers (*n* = 20) are designated to stay “COVID-19–free”. The German Heart Center Berlin [Deutsches Herzzentrum Berlin (DHZB)] is a tertiary cardiovascular center and classified as level III. In addition to this allocation, all hospitals were required to postpone elective treatments and to increase the number of immediately available intensive care unit (ICU) beds.

As there is a lack of data on the prevention of in-hospital infections with SARS-CoV-2 in patients and HCWs, the purpose of this report is to describe the combined effect of hospital-initiated measures in addition to governmental regulations during the early phase of the COVID-19 pandemic in Berlin.

## Methods

The study was approved by the local ethics committee (no. EA2/092/20, PREV-SARS-CoV-2-DHZB) and was performed in accordance to the declaration of Helsinki. Human studies are presented. Informed consent was obtained from all participants orally and in writing.

The German Heart Center Berlin is a specialized hospital for the treatment of cardiovascular diseases (cardiothoracic surgery for adults and children, cardiology, pediatric cardiology, anesthesiology), which treated >8,300 inpatients and >25,500 outpatients in 2019 employing a staff of 1,404 people.

**Figure 1:**
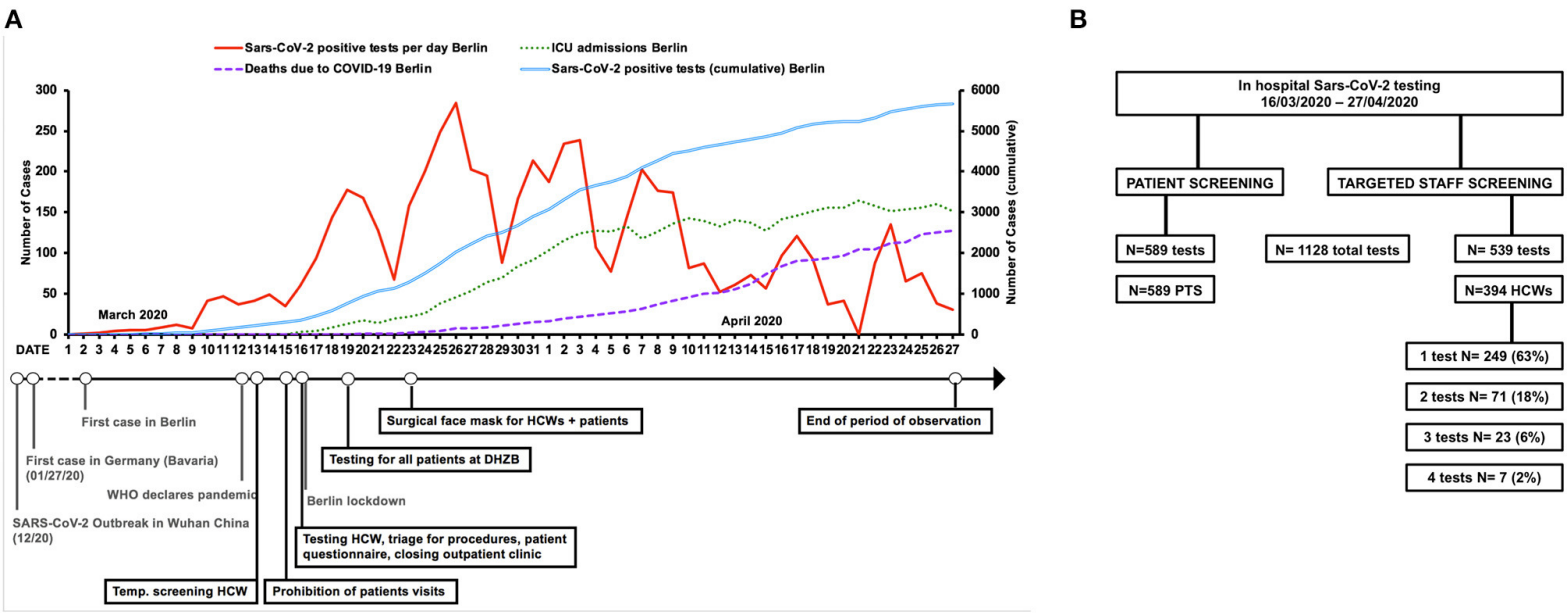
Timeline of the COVID-19 pandemic in Berlin and preventive measures initiated by the German Heart Institute (DHZB). **(A)** Timeline of COVID-19 pandemic and in-hospital SARS-CoV-2 preventive measures. Upper part: positive SARS-CoV-2 tests per day (red line) and cumulative SARS-CoV-2 tests (blue line), cumulative number of ICU admissions (green dotted line), and deaths due to COVID-19 (dotted purple line) in Berlin. Lower part: timeline and key measures initiated during the SARS-CoV-2 pandemic (gray: national and worldwide actions, black: DHZB measures). **(B)** Flowchart of SARS-CoV-2 RT-PCR testing at the German Heart Institute Berlin (DHZB) during the period of 03/16/2020–04/27/2020 in healthcare workers (HCWs) and patients (PTS). HCWs, healthcare workers; DHZB, Deutsches Herzzentrum Berlin (German Heart Center Berlin); RT-PCR, real-time polymerase chain reaction; PTS, patients; Temp, temperature.

During the time of this study (03/16/2020–04/27/2020), several recommendations and rules were initiated by German and local government agencies to contain the spread of COVID-19. Figure 1A depicts the timeline of measures initiated by German/state authorities and the German Heart Center in relationship to the COVID-19 pandemic.

### Measures Initiated by Government and State Agencies

By 03/12/2020, the German government and local authorities decided to cancel major events of more than 1,000 people, to postpone elective medical procedures and increase ICU capacities. On 03/13/2020, 14 of the 16 German federal states decided to close their schools and nurseries, including Berlin. Visits to nursing homes and hospitals were prohibited. Contact restrictions were expanded on 03/22/2020, with gatherings of more than two people banned and a required minimum physical distancing of 1.5 m in public (19).

### Measures Initiated by the German Heart Center

The following measures were initiated: standard operating procedures focusing on patient admission/treatment and protection of patients/employees from SARS-CoV-2 infections were implemented. From 03/13/2020, twice daily temperature screening for HCWs was done. From 03/15/2020, all visitors were prohibited, except for pediatric patients <16 years of age (maximum one parent). From 03/16/2020, patient risk stratification/triage for planned procedures/operations was initiated based on disease, symptoms, and comorbidities, and non-emergent medical/surgical treatments were postponed. Upon hospital admission, patients underwent a questionnaire survey including symptoms, contacts to COVID-19, and travel history. Outpatient departments were closed for routine visits. Routine testing of all patients for SARS-CoV-2 infection was started on 03/19/2020. Universal in-hospital masking (surgical masks; employees; and patients) was obligatory from 03/23/2020 on hospital premises. From 03/16/2020, a voluntary testing was offered to all employees in case of a suspected SARS-CoV-2-infection (Figure 1A).

### SARS-CoV-2 Testing

As shown in Figure 1B, 1,128 SARS-CoV-2 polymerase chain reaction (PCR) tests were performed in 983 individuals during the period of this study (589 tests in patients, with all patients tested and 539 tests in 394 employees). Indications for testing were different for patients and hospital employees. All patients admitted to the hospital were routinely tested for SARS-CoV-2. Whenever possible, the test was administered by the patient themselves as a swap from the posterior wall of the oropharynx, which was successfully done in >95% of cases. Patients were given standardized instructions from a nurse along with a visual aid for self-collection. If the patient needed assistance, the test was performed by an HCW using adequate personal protective equipment (PPE). In case of a positive test, the patient was isolated and transferred to a COVID-19–designated hospital, a contact list compiled, and reporting to health authorities done. Contacts were tested for SARS-CoV-2.

Voluntary testing was offered to 1,404 clinical and non-clinical employees including 199 physicians and 383 nurses (= clinical workforce). Testing was offered in case of illness, but also to asymptomatic employees who returned from risk areas, had contact to a SARS-CoV-2 positive person, or had close contact to a person who had contact to a COVID-19 patient. The test was administered by the employees as a swap from the posterior wall of the oropharynx with a visual aid provided. A questionnaire documented reason/motivation for testing (i.e., contact, symptoms, risk area travel). Symptoms were specified as follows: fever, dry cough, productive cough, fatigue, shortness of breath, jaw pain, sore throat, headache, chill, nausea, general malaise, myalgia, rhinitis, diarrhea, and stuffed nose. In case of a positive test result, quarantine was ordered, and a contact list done. Contacts were tested for SARS-CoV-2.

### SARS-CoV-2 Real-Time PCR

Swab collections were performed with identical test material (flocked swab, transport tube with 2–3 mL of viral transport medium). Three different systems for SARS-CoV-2 RNA-detection were used, based on prioritization: tests on patients with highly urgent treatment indication were performed using the Xpert® Xpress SARS-CoV-2 (Cepheid, Sunnyvale US), a cartridge-based system that provides results for SARS-CoV-2-RNA detection in <1 h. Other testing was performed on the BD-MAX™ System (Becton Dickinson, Franklin Lakes, US) using VIASURE SARS-CoV-2 RT-PCR reagents (Certest Biotec, Zaragoza, Spain), with a test duration of 2.5 h. These two systems are available on-site. In case of insufficient capacity, tests are additionally performed at the Medizinische Infektiologiezentrum Berlin. In this off-site location, tests were done on a Seegene Inc. Nimbus IVD system using the Allplex™ 2019-nCoV Assay on a Bio-Rad CFX96 Real-Time-PCR cycler with a test duration of 4.5 h. Test performance of all systems was shown to be identical. All laboratory sites are accredited by the Deutsche Akkreditierungsstelle GmbH (DAKKs) for performing molecular testing on viral pathogens. All assays used are CE/IVD-marked, and test performance was evaluated using positive patient samples and samples from External Quality Assessment (EQA) Panels including successful participation in EQA trials with all used systems.

### Statistical Analysis

We retrospectively analyzed data of a 6-week observational period from 03/16/2020 to 04/27/2020. Continuous variables are described by mean ± standard deviation or median (minimum–maximum or interquartile range), respectively. After testing for normal distribution by Shapiro–Wilk test, group comparisons were performed by using Student *t*-test or Mann–Whitney *U*-test. Categorical variables are presented in absolute numbers and relative frequencies, group comparisons were performed by using the Pearson χ^2^-test. Odds ratios (ORs) and confidence interval (CI) were calculated by logistic regression. Throughout all calculations, a two-tailed probability *P* < 0.05 indicated statistical significance. Statistical analysis was conducted using SPSS version 26 (SPSS Inc., Cary, NC, USA).

## Results

The DHZB has a total of 1,404 employees including 199 physicians and 383 nurses.

Figure 1A depicts the hospital-initiated measures in relationship to restrictions by German and local authorities, as well as their temporal correlation to the number of positive SARS-CoV-2 tests, COVID-19 ICU admissions, and COVID-19–related deaths in Berlin. Overall, 1,128 SARS-CoV-2-PCR tests were done in 983 individuals during the period of this study. Of these, 589 tests were done in patients, with all patients (100%) undergoing one single test. In contrast, 394 employees did 539 tests, with 37% receiving more than one test (mean, 1.37 test/employee). The majority of HCWs had one test (72%, *n* = 286), 20% had two tests (*n* = 78), 6% had 3 tests (*n* = 23), and 2% had 4 tests (*n* = 7) (Figure 1B). In total 28.1% of hospital employees were tested. With regard to the clinical staff, we tested 57% (mean: nursing staff 60%, doctors 54%).

### Symptom-Based SARS-CoV-2 Testing in Hospital Employees

Characteristics of employees tested for SARS-CoV-2 are shown in Table 1. More females (65%, *n* = 256) than males (35%, *n* = 138) were tested. The median age was 42 years [range, 19–71 years; interquartile range (IQR), 42–53 years]. One hundred fifty-nine of the tests were done in physicians (29%), 319 in nurses (59%), and 61 (11%) in persons from other work areas (i.e., mechanics, administration). Accordingly, the rate of tests in individual employees done per profession was 54% in physicians (*n* = 107 of 199), 60% in nurses (*n* = 231 of 383), and 7% in other work areas (*n* = 56 of 822). The majority of tests were done during the first 2 weeks of the observation period (up to 44 tests/day on 03/19/2020).

**Table 1:** Baseline characteristics of hospital employees tested for SARS-CoV-2.

**Variables**	**Overall (*n* = 394)**
Median age, years (range)	42 (19–71)
Female	256 (65)
Physicians	107 (27)
Nurses	231 (59)
Others	56 (14)
No. of tests, mean (median)	1.35 (1)

*Values are given as n (%), mean or median (range)*.

**Figure 2:**
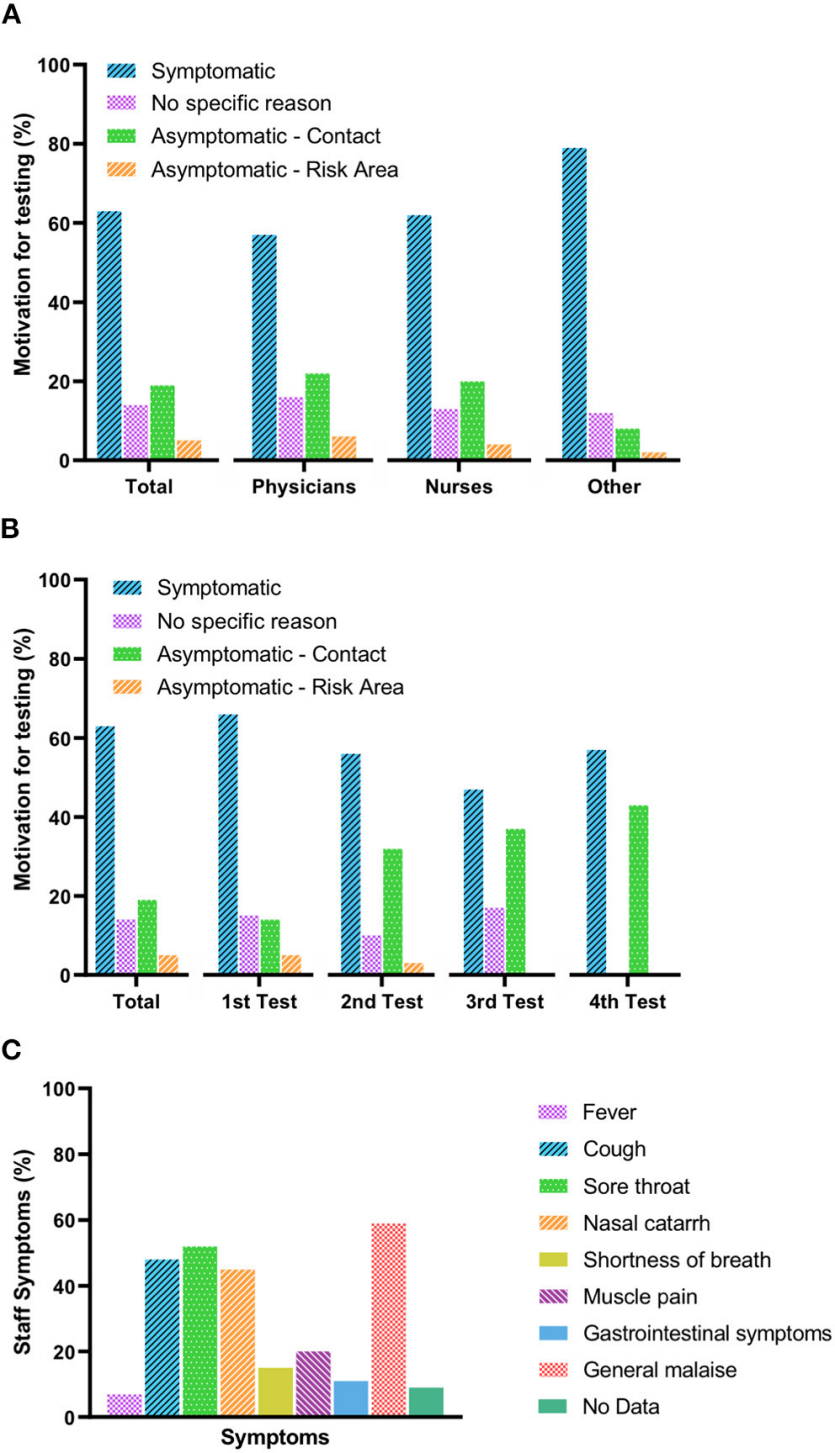
Motivation for SARS-CoV-2 testing and symptoms reported by healthcare workers. **(A)** Motivations (percentage) for SARS-CoV-2 testing per professional group. **(B)** Motivations (percentage) for SARS-CoV-2 retesting in healthcare workers. **(C)** Symptoms reported by healthcare professionals. Fever is defined as body temperature over 38°C; cough includes dry and productive cough; nasal catarrh includes rhinitis and stuffed nose; muscle pain includes general and jaw muscle pain; gastrointestinal symptoms include diarrhea, nausea, and vomiting; and general malaise includes fatigue, headaches, chill, and general discomfort.

Hospital employee's motivation for undergoing SARS-CoV2-testing is shown in Figures 2A,B. Most tests (62.5%, 337 of 539 tests) were done due to the development of symptoms (*P* < 0.01), whereas 202 of 539 tests (37.5%) were done in asymptomatic employees. Of tests done in the asymptomatic employees, 4.5% (*n* = 24) were in returnees from risk areas, 19.3% (*n* =104) in employees reporting contact to a COVID-19 patient (direct or close indirect contact), and 13.7% (*n* = 74) in asymptomatic employees without any contact, symptoms, or risk-area stay. Symptoms as motivation for testing was significantly more often denoted by non-physicians and non-nursing staff as compared to nurses (*P* = 0.015; OR, 2.23; CI, 1.16–4.28) and physicians (*P* = 0.003; OR, 2.83; CI, 1.42–5.64). Contact to a confirmed case of COVID-19 was denoted significantly less often by non-HCWs as compared to nurses (*P* = 0.028; OR, 0.37; CI, 0.14–0.92) and physicians (*P* = 0.018; OR, 0.32; CI, 0.12–0.9; Figure 2A). Even if more than one test was done, symptoms remained the driving force (first test = 66%, *n* = 259; second test = 56%, *n* = 60; third test = 47%, *n* = 14; fourth test = 57%, *n* = 4), but the percentage of asymptomatic HCWs that requested testing due to contact with an (presumptively or confirmed) infected person increased (first test = 14%, second test = 32%, third test = 37%, fourth test = 43%). Contact to a confirmed case of COVID-19 was denoted significantly less often in the first compared to second test (*P* < 0.001; OR, 2.77; CI, 1.69–4.55), third test (*P* = 0.001; OR, 3.49; CI, 1.58–7.74), and fourth test (*P* = 0.034; OR, 4.53; CI, 0.99–20.77). Risk-area return was a rare initial reason (5%, *n* = 21; Figure 2B). Testing performed in employees was accompanied by a questionnaire (multiple symptoms possible). The four most common symptoms reported were general malaise (59%, n = 200), sore throat (52%, *n* = 176), cough (48%, *n* = 163), and nasal catarrh (45%, *n* = 153; Figure 2C). Myalgia was reported in 20% (*n* = 68), shortness of breath in 15% (*n* = 52), and gastrointestinal symptoms in 11% (*n* = 38). In contrast, fever was indicated in only 7% (*n* = 25). In 9% (*n* = 30), employees stated being symptomatic, but did not specify any other reason (Figure 2C).

Of the 539 tests performed in 394 employees, only 3 (0.8%) tested positive for SARS-CoV-2. Two positive results occurred in first-time participants. The characteristics of positive HCWs are outlined in Table 2. Two of them had no comorbidity; one reported hypertension and bronchial asthma. The suspected source of infection was community acquired in all cases (one returnee from a risk area, one indirect contact via children's school, and one at a medical conference). Two of the three HCWs made use of testing because of mild symptoms (Table 2). The third HCW was initially asymptomatic, but underwent testing because of travel return from Ischgl/Austria. Her initial test was negative, but her fiancé tested positive. Thus, SARS-CoV-2 testing was redone 4 days later and was positive. She reported anosmia, but none of the three staff members reported fever.

**Table 2:** Characteristics of healthcare workers tested positive for SARS-CoV-2.

**Variables**	**HCW 1 (physician)**	**HCW 2 (nurse)**	**HCW 3 (nurse)**
Age (years)	40	30	50
Sex	Male	Female	Female
Comorbidities	None	None	Arterial hypertension Bronchial asthma
Symptoms	Myalgia Dry cough Headaches	Initially Asymptomatic Anosmia	Myalgia Anosmia Headaches
Suspected source of infection	Dyspnea Medical conference	Risk area (Ischgl/Austria)	Contact (indirect)
In-hospital contacts	12	7	25
Contacts tested	12 (100)	7 (100)	24 (96)
Tested positive for SARS-CoV-2	0	0	0

*Values are given as n (%). HCW, healthcare worker*.

### Obligatory SARS-CoV-2 Testing in Hospitalized Patient

All 589 inpatients (100%) admitted were tested for SARS-CoV-2 at admission. Patients were mostly male (69.1%, *n* = 407; female: 30.9%, *n* = 182), with a median age of 64 years (range, 0–90 years; IQR, 49–74 years). Of the 589 tests performed, 58% (*n* = 342) were done in the department of cardiothoracic surgery, 30.1% (*n* = 177) in the department of medicine/cardiology, and 11.9% (*n* = 70) in the department of pediatric cardiology.

**Figure 3:**
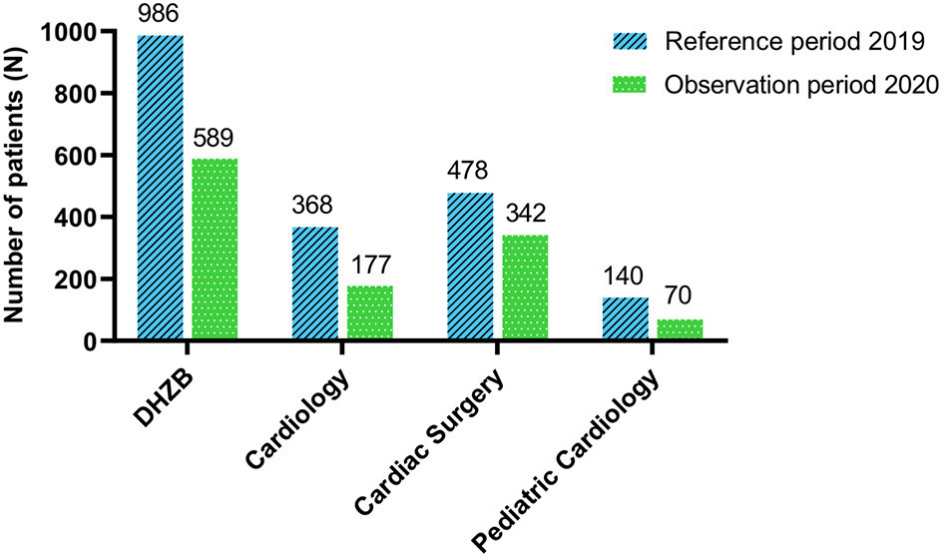
Number of inpatients treated during the COVID-19 pandemic in comparison to the reference period in 2019. Total reduction of patients admitted during the reported period compared to the same time period in 2019 in different departments of the German Heart Center Berlin. DHZB, Deutsches Herzzentrum Berlin (German Heart Center Berlin).

In 2019, the DHZB treated 8,378 inpatients and 23,523 outpatients. During the observation period, the DHZB treated 40.3% less inpatients compared to the corresponding period in 2019 (2019 *n* = 986 patients vs. 2020 *n* = 589 patients; Figure 3). Patients treated in the department of medicine/cardiology (*n* = 165) were further analyzed with respect to their comorbidities and compared to patients in 2019. During the surge of COVID-19, patients admitted had significantly more cardiovascular risk factors (3.50 vs. 3.09, *P* < 0.02), significantly more heart failure (52.7 vs. 37.1%, *P* < 0.001), and a significant decrease in left ventricular systolic ejection fraction (48.2 vs. 52.7%, *P* < 0.001). In addition, valvular heart disease was significantly more present (41.2 vs. 29.7%, *P* = 0.01). Neither age, body mass index, nor the diagnosis of coronary artery disease, peripheral artery disease, or chronic obstructive lung disease was different (Table 3).

**Table 3:** Baseline characteristics of patients in the department of medicine/cardiology during the reference period in 2019 and the study period.

**Variables**	**Reference period (2019) (*n* = 377)**	**Study period (2020) (*n* = 165)**	***P*-value**
Mean age, years	70 ± 12.5	68 ± 12.6	0.68
Mean LVEF, %	52.7 ± 12	48.2 ± 13.8	<0.001
Mean BMI, kg/m^2^	28.09 ± 5.7	27.60 ± 5.2	0.10
Mean number of CVRF	3.09 ± 1.3	3.50 ± 1.5	0.02
Heart failure	140 (37.1)	87 (52.7)	<0.001
Coronary artery disease	243 (64.5)	102 (61.8)	0.56
Valvular heart disease	112 (29.7)	68 (41.2)	0.01
Peripheral artery disease	41 (10.9)	14 (8.5)	0.44
COPD	37 (9.8)	19 (11.5)	0.54

*Values are given as n (%), mean ± standard deviation. Reference period: 03/16/2019–04/27/2019, study period: 03/16/2020–04/27/2020. LVEF, left ventricular ejection fraction; BMI, body mass index; CVRF, cardiovascular risk factors (arterial hypertension; hypolipoproteinemia, diabetes mellitus, smoking, family history of cardiovascular disease); COPD, chronic obstructive pulmonary disease*.

Of the 589 patients screened for SARS-CoV-2, only two patients tested positive (0.3%; one female and one male). One of the patients was asymptomatic with respect to COVID-19 and admitted for valvular heart surgery; the other one had pulmonary symptoms, fever, and diarrhea, and COVID-19 was suspected. As this patient was a heart transplant recipient and presented as emergency, he was admitted and isolated. None of the patient's contacts (mean *n* = 15) was infected. Detailed characteristics of the patients are shown in Table 4.

**Table 4:** Individual characteristics of the two patients tested positive for SARS-Cov-2.

**Variables**	**Patient 1**	**Patient 2**
Age (years)	72	53
Sex	Female	Male
Comorbidities	Hypertension CAD (CABG) Heart failure Valvular heart disease Chronic aortic dissection Thoracic aortic aneurysm Surgery	Heart transplant Hypertension DCM CKD (hemodialysis) Thoracic aortic aneurysm Surgery
Symptoms	Dyspnea (overlap to underlying disease)	Fever (38.8°C) Productive cough Rhinitis Dyspnea Diarrhea
Date of first positive test result	27/03/2020	20/04/2020
Days after first confirmed case in Germany/Berlin	60/25	84/49
Suspected source of infection	Unknown	Unknown
Contacts	8	22
Contacts tested positive for SARS-CoV-2	0	0

*Values are given as n (%). CAD, coronary artery disease; CABG, coronary artery bypass grafting; DCM, dilative cardiomyopathy; CKD, chronic kidney disease*.

## Discussion

We report interventions undertaken by a major cardiovascular center to prevent nosocomial patient and hospital employee SARS-CoV-2 infection, resulting in a low overall infection rate of 0.5%. Our data focus on the time span in which a number of restrictions were initiated (03/12/2020) by German and regional authorities (e.g., closure of schools, physical distancing) due to the exponential up rise of the COVID-19 pandemic and ends when these restrictions were partly lifted (e.g., reopening of schools and retail) due to lessening of the infection rates (19, 20). Restrictions were escorted by several hospital-initiated measures, including the review of scheduled visits for urgency and postponing elective operations, as well as closure of outpatient departments. However, in contrast to health authorities who did not recommend wearing a surgical face mask or screening for SARS-CoV-2 infection in patients and HCWs during that time, we initiated both at the very early beginning of the pandemic (21). Furthermore, symptom-based staff testing (03/16/2020) and mandatory patient testing (03/19/2020) were initiated early on.

During this observation period, the number of positive SARS-CoV-2 tests sharply increased in Germany and Berlin and was paralleled by an increase in COVID-19 ICU admissions and deaths. The reported positive tests/day rate for Germany was 6.8% at the beginning and declined to 3.9% at the end of our study period (22). However, we found only three HCWs (0.8%) and two patients (0.3%) infected with SARS-CoV-2, despite the fact that we screened 100% of patients and up to 60% of the clinical workforce (nurses and physicians).

Healthcare workers are at an increased risk of SARS-CoV-2 exposure, but may also be the source of nosocomial infections for patients and coworkers (15, 23). Early in the course of the pandemic, a single-center study from a large tertiary hospital in Wuhan, China (>7,000 beds), reported an infection rate of 0.5% in “first-line” HCWs, which was mostly hospital-acquired (15). Interestingly in this study, first-line HCWs working in close contact to COVID-19 patients had a lower infection rate than HCWs working in other clinical departments (1.6%), likely due to a better adherence to the use of PPE (15). More recently, a study from seven community hospitals in Texas reported the opposite, with 5.4% HCWs from COVID-19 units being SARS-CoV-2-positive, but only 0.6% from non-COVID-19 units (24). For the United States, the Centers of Disease Control and Prevention states that up to 55% of infected HCWs had contact with a COVID-19 patient solely in a healthcare environment, suggesting that work-related COVID-19 is common in HCWs (16). In contrast, in the Netherlands, SARS-CoV-2 infection among HCW was reported to be mostly community acquired (25). For Germany, data from a national survey reported a total of 495 COVID-19 outbreaks in hospitals/rehabilitation facilities across the country, resulting in 5,225 infections (26). At least 7% of SARS-CoV-2–infected persons were working in a medical setting in Germany during the first wave (27).

Here we describe the initiation of measures initiated at the same time, which may have worked in concert. First, this involves the designation of our hospital as “non–COVID-19” hospital, and like others, we postponed non-urgent cases, significantly reducing the number of patients by 40% (28, 29).

Second, with regard to SARS-CoV-2 testing, in this report, we investigated different hospital populations (patients vs. employees) by different modes (obligatory vs. symptom-based) of testing. Both groups likely differ by risk behavior, with cardiovascular patients at older age presumptively practicing more physical distancing during the pandemic. In Germany and other countries, the pandemic is mostly driven by the younger/middle-age working population (30). Strikingly, the infection rate in this age group is low in our hospital. However, we did not screen all employees for SARS-CoV-2 and may have missed asymptomatic/presymptomatic infected. The viral load in asymptomatic and symptomatic patients is comparable, and transmission of SARS-CoV-2 by atypical/presymptomatic individuals has been shown to cause clusters of cases in defined sectors (8, 13, 31). Data on the numbers of asymptomatic infected persons vary significantly, ranging from 1% in early publications from China to more than 10% in a population-based study in individuals in Iceland (12, 32, 33). In contrast to our ubiquitous patient testing, we had to use a symptom-based approach for employee testing, because of limited resources. Symptoms mostly reported in our study included general malaise, sore throat, cough, and nasal catarrh. Still, a recent report demonstrated the limitation of symptom-based screening: when fever, cough, shortness of breath, or sore throat were asked, up to 17% of SARS-CoV-2–infected cases were missed, and even when expanding these criteria to include myalgias and chills, 10% were still missed (34). Thus, ubiquitous staff testing would have been desirable.

Indeed, a number of reports demonstrated that COVID-19 outbreaks can result from single index cases (13, 31, 35). A detailed epidemiological/phylogenetic study from South Africa showed that one SARS-CoV-2–infected person led to clusters in different hospital wards, leading to 39 infected patients and 80 infected staff members (35). Likewise, a recent report from a German teaching hospital demonstrated that only one index COVID-19 patient led to five infected staff members, subsequently resulting in more than 30% of infected hospitalized patients, emphasizing the need for a widespread SARS-CoV-2 testing and rapid isolation of positive cases (14). Thus, it is imperative to provide a safe hospital environment for patients and employees.

Third, in addition to widespread testing, studies now demonstrate that in contrast to early advice from health authorities, face masks are not a substitute, but significantly impact on SARS-CoV-2 transmission by protecting others from infected droplets (23, 36–39). A study performed in the largest healthcare system in Massachusetts (12 hospitals, >75,000 employees) demonstrated that prior to universal masking of HCWs and patients, new infections among HCWs sharply raised from 0 to 21.3% (39). Following mandatory face masking for patients and staff (among other restrictions), the positivity rate decreased linearly down to 11.46% (39). Another study done at Duke Health in North Carolina, US (>20,000 HCWs, including a tertiary care facility, community hospitals, primary care, and specialty practices) reported an analysis in which 70% of healthcare-associated SARS-CoV-2 infections were related to unmasked exposure to another HCW and only 30% secondary to direct care of SARS-CoV-2-positive patients (23).

Even though not randomized trials, these studies and our present report, in which we initiated surgical face masking for patients and HCWs at the very beginning, support that this simple intervention in combination with testing for SARS-CoV-2 is a key means to prevent COVID-19 in-hospitals outbreaks.

### Study Limitations

We used self-administered oropharyngeal swaps instead of HCW-administered nasopharyngeal specimen collection. This lowers the risk of infection for the clinical staff and saves PPE resources. Indeed, studies demonstrated that swaps from different clinical specimens are comparable and that collection of patient samples for SARS-CoV-2 testing is accurate and valid (40, 41). Therefore, it is unlikely that this affected the results of our observation. Another limitation is that we could not provide universal screening to all employees because of limited testing resource. In addition, we report a single-center, non-interventional study that might not represent all healthcare systems/providers across Germany/Europe.

## Data Availability Statement

The raw data supporting the conclusions of this article will be made available by the authors, without undue reservation.

## Ethics Statement

The study was approved by the institutional ethics committee (Ethics Committee of the Charité - Universitätsmedizin Berlin; number: EA2/092/20; acronym: PREV-Sars-CoV-2-DHZB) and was performed in accordance with the ethical standards as laid down in the 1964 Declaration of Helsinki and its most recent amendment 2013. Informed consent was obtained from all participants orally and in writing according to the Helsinki Declaration.

## Author Contributions

DS, KW, ME-M, MH, OM, FS, SH, and PS contributed to conception, design of the work, contributed to analysis, and interpretation of the data for the work. DS, KW, and PS drafted the manuscript. MH, OM, FS, SH, and BP critically revised the manuscript. All authors gave the final approval and agree to be accountable for all aspects of work ensuring integrity and accuracy.

## Conflict of Interest

DS received travel grants from St. Jude Medical, Bristol-Myers-Squibb and Biosense Webster, a research grant from Biosense Webster and took part in the Boston scientific EP fellowship program. FS reports non-financial support from Medtronic, grants from Novartis, grants from Abbott, personal fees from Cardiorentis, outside the submitted work. BP reports having received consultancy and lecture honoraria from Bayer, Daiichi Sankyo, MSD, Novartis, Sanofi-Aventis, Stealth Peptides and Vifor Pharma; and editor honoraria from the Journal of the American College of Cardiology. PS has received consultancy and lecture honoraria from Amgen, Novartis, Sanofi-Aventis, Bristol-Myers Squibb/Pfizer, Daiichi-Sankyo, Bayer, Boehringer Ingelheim, BerlinChemie, B. Braun, Medtronic, AstraZeneca and editor honoraria from Springer Nature. The remaining authors declare that the research was conducted in the absence of any commercial or financial relationships that could be construed as a potential conflict of interest.
